# Leptin Induced TLR4 Expression via the JAK2-STAT3 Pathway in Obesity-Related Osteoarthritis

**DOI:** 10.1155/2021/7385160

**Published:** 2021-08-17

**Authors:** Mengqi Jiang, Jianyi He, Yingxu Sun, Xin Dong, Jiayu Yao, Hailun Gu, Li Liu

**Affiliations:** ^1^Department of Nutrition and Food Hygiene, School of Public Health, China Medical University, 110122, China; ^2^Department of Orthopedics, Shengjing Hospital, China Medical University, 110004, China

## Abstract

Obesity is considered as a risk factor of osteoarthritis (OA), but the precise relationship is still poorly understood. Leptin, one of the most relevant factors secreted by adipose tissues, plays an important role in the pathogenesis of OA. Our aim was to investigate the regulation and molecular mechanism of the leptin signaling pathway in obesity-related OA. SD rats were fed with a high-fat diet (HFD) for 5, 15, and 27 weeks. The levels of leptin in serum increased from W5, while in the synovial fluid increased from W15. The histological evaluation showed that the pathological changes of OA occurred at 27 weeks rather than 5 or 15 weeks. We also found that leptin induced CD14/TLR4 activation by the JAK2-STAT3 signaling pathway to promote OA. Moreover, silencing SOCS3 enhanced leptin-induced JAK2-STAT3-CD14/TLR4 activation in rat primary chondrocytes. Our findings indicated that leptin may be one of the initiating factors of obesity-related OA. TLR4 is at least partially regulated by leptin through the JAK2-STAT3-CD14 pathway. Meanwhile, SOCS3 acting as a negative feedback inhibitor of leptin signaling presented a potential therapeutic prospect for obesity-related OA. Our study provided new evidence suggesting the key role of leptin in mediating obesity-related OA process and its underlying mechanisms.

## 1. Introduction

Osteoarthritis (OA) is one of the most common musculoskeletal disorders that affect millions of individuals. It is characterized structurally by degradation of the articular cartilage and subchondral bone remodeling, accompanied by joint malfunction, chronic pain, and loss of function [1]. There are a number of risk factors that are related to OA, among which obesity-related OA has attracted more and more attention.

Obesity increases the mechanical load of weight-bearing joints and promotes the degeneration of the articular cartilage. However, the joint load is not the only cause of obesity-related OA. Chronic low-grade systematic inflammation caused by obesity plays a significant role in obese OA, and the cytokine leptin mainly secreted by excessive adipose tissues in obese people is considered to be a causative link between obesity and OA [2]. Leptin is a peptide hormone which was originally reported to play an important role in energy metabolism by the reason that it may cause a loss of appetite and increased energy expenditure [3]. Because of the higher serum levels in overweight people and the joint symptoms reduction by reducing weight in patients with OA, leptin may be presumed to be a necessary factor for obesity-related OA. It is worth noting that Griffin et al. proved that a lack of leptin does not lead to an increase in the incidence of spontaneous OA in a mouse model, although its body weight is extremely high, indicating that the loss of leptin signaling may protect the human body from the progress of OA [4], which has drawn our attention whether leptin plays an initial role in the pathogenesis of obesity-related OA or it is just a driver of OA progress, and by what mechanism does leptin promote OA.

Toll-like receptor (TLR) 4, as a member of the natural immune receptor family, is considered to be the most important TLR for obesity-related inflammatory responses. Vitseva et al. reported that TLR4 can be used as an endogenous lipid and fatty acid sensor to further regulate metabolism and the immune system [5]. Triglycerides can activate the TLR4 signaling pathway in hepatocytes and increase the expression of inflammatory factors [6]. It may infer that low inflammation triggered by obesity is closely related to the expression of TLR4. Studies have shown that the expression of TLR4 and its downstream signaling pathway in the articular cartilage of obese OA mice induced by a high-fat diet are significantly increased; meanwhile, the expression of TLR4 is positively correlated with serum leptin concentration [7]. Additionally, although high-fat diet can induce weight gain in TLR4 knockout mice, there is no inflammation in endothelial vessels [8], while the expression of the TLR4 gene in visceral fat of obese ob/ob mice with leptin mutation is significantly lower than that of obese mice induced by high-fat diet [9]. It may indicate that TLR4 is involved in inflammatory regulation, and leptin regulates the expression of TLR4.

Studies have found that leptin activates the cluster of differentiation 14 (CD14) expression via the Janus kinase 2/signal transducer and activator of transcription 3 (JAK2/STAT3) signaling pathway [10], and the pathological activation of the CD14/TLR4 pathway is involved in the development of various inflammatory and rheumatic diseases. It can regulate the apoptosis of articular chondrocytes and mediate the damage of progressive extracellular matrix and the degradation of the articular cartilage [11]. Therefore, leptin may activate CD14 via the JAK2/STAT3 signaling pathway to regulate TLR4 expression. The suppressor of the cytokine signaling (SOCS) family is a group of proteins induced by cytokines discovered in recent years, with SOCS3 being the most active member. SOCS3 is induced by leptin-JAK2-STAT3 signaling and is the most important negative feedback regulator of this signaling pathway [12]. In the study of cartilage, the biological effects of leptin were also regulated by SOCS3, and leptin-induced catabolic effects in chondrocytes with low expression of SOCS3 were enhanced [13]. In the occurrence and development of obesity-related OA, SOCS3 may be a key factor in the regulation of TLR4 signaling by leptin.

Thus, in this study, we explored the regulation and molecular mechanism of the leptin signaling pathway in obesity-related OA. The data presented in this work showed that leptin may be one of the initiating factors of obesity-related OA. Leptin induces CD14/TLR4 activation by the JAK2-STAT3 signaling pathway to promote OA. Meanwhile, the negative feedback effect of SOCS3 on leptin signaling in the cartilage is relatively weak, which provided new evidence indicating the key role of leptin in mediating OA process and its underlying mechanisms.

## 2. Material and Methods

### 2.1. Animals and Treatments

We got 6-week-old male SD rats from Laboratory Animal Center of China Medical University. After 2 weeks of adaptive feeding, the rats were randomly divided into the CON group (*n* = 36, fed a control diet with 10% of the calories from fat) or the HFD group (*n* = 36, fed a high-fat diet with 60% of the calories from fat). In each group, diet started from 8 weeks old and sustained for 5 weeks, 15 weeks, and 27 weeks (*n* = 12 each). All rats were housed at normal ambient room temperature (22 ± 2°C) under a 12 h-light-dark cycle condition with free access to food and water, and the body weight was monitored weekly. All animal experimental operations were carried out according to the Basel Declaration and recommendations of Guide for the Care and Use of Laboratory Animals, China Medical University Institutional Animal Care and Use Committee (20180228-48).

### 2.2. Histological Evaluation

Whole knee joints of rats were fixed in 4% paraformaldehyde, decalcified, and embedded in paraffin, and then sectioned for histological and immunohistochemistry evaluation. The sections were stained with Safranin O (Solarbio, Beijing, China), H & E (Solarbio, Beijing, China), or Safranin O/Fast Green (Solarbio, Beijing, China) to determine the histological change of the cartilage. The severity of cartilage destruction was assessed by modified Mankin scores [14].

### 2.3. Immunohistochemistry

The deparaffinized slides were rehydrated in gradient concentrations of ethanol and rinsed out in PBS. The slides were incubated in a sodium citrate solution buffer to perform antigen retrieval. Then, the slides were incubated with 0.3% hydrogen peroxide for 30 min at room temperature. The slides were then incubated with anti-matrix metalloprotease-13 (MMP-13) IgG (Abcam, USA) at a dilution of 1 : 200. After an overnight reaction with the primary antibody at 4°C, the slides were incubated with HRP antirabbit IgG (Abcam, USA) at room temperature. Each slide immunostained for MMP-13 was taken with a light microscope (Nikon, Japan). The percentages of positive-stained cells for MMP-13 were shown by ImageJ 2x software. All immunostaining values were obtained from six randomized fields for statistical analysis.

### 2.4. Measurement of Serum and Synovial Fluid Leptin

The leptin levels in the serum and synovial fluid (SF) were evaluated by an ELISA kit obtained from Boster (Wuhan, China), in line with the manufacturer's instructions.

### 2.5. Real Time PCR

Total RNA was extracted from the cartilage using TRIzol reagent (Sangon Biotech, Shanghai, China). The RNA purity and concentration were measured using NanoDrop 2000 spectrophotometer (Thermo, USA). The cDNAs were synthesized using PrimeScript RT-PCR System kit (CwBio, Inc., Beijing, China) and amplified using SYBR Green Master Mix (TaKaRa, Dalian, China) on Q6 Real-Time PCR System (Thermo, USA). All primer sequences used in this study are as follows: *β*-actin (F) 5′-CACACTGTGCCCATCTACGA-3′, (R) 5′-CTCAGTGAGGATCTTCATGAGGTAGT-3′; CD14 (F) 5′-TGGCCCAGTCAGCTAAACTC-3′, (R) 5′-AGGGTTCCTATCCAGCCTGT-3′; and TLR4 (F) 5′-AGGACTGGGTAAGGAATGAGC-3′, (R) 5′-ATCACCTTTCGGCTTTTATGG-3′. 𝛽-Actin was used as an internal control to calculate the relative expression of genes (2^−*ΔΔ*Ct^ method).

### 2.6. Western Blot

The total proteins were extracted using the protein lysis buffer (Sangon Biotech, Shanghai, China). The concentration of extracted proteins was measured using BCA kit (Dingguo Bio, China). The extracted proteins were loaded onto 8-15% SDS/PAGE gradient gels, and then, the protein samples were transferred onto a PVDF membrane. After blocking with 5% skimmed milk at room temperature for 2 h, the membrane was incubated overnight at 4°C with primary antibodies against JAK2, P-JAK2, STAT3, P-STAT3 (dilution 1: 1000, CST, USA), CD14, MMP-13 (dilution 1 : 1000, Proteintech Group, Wuhan, China), TLR4, SOCS3, and *β*-actin (dilution 1: 1000, Santa Cruz, CA, USA) and then incubated with HRP-conjugated secondary antibody (1 : 5000 dilution, Abclonal, Wuhan, China) at room temperature for 1 h. The band images were developed by Super ECL Reagent (Millipore, MA, USA) and analyzed by ImageJ 2x software.

### 2.7. Cell Culture and Treatments

Primary chondrocytes were isolated from newborn SD rats as described previously [15]. Briefly, the articular cartilages of rats were dissected and dissociated, and the cells were cultured using DMEM/F12 medium with 10% fetal bovine serum at 37°C and 5% CO_2_. Primary chondrocytes were incubated in six-well microplates at 37°C and 5% CO_2_ in a humidified chamber for 24 h. Then, cells were pretreated with or without 10 *μ*M JAK2/STAT3 inhibitor AG490 (T3434, Sigma, Saint Louis, USA) for 2 h in combination with or without 200 ng/mL leptin (R&D, MN, USA) stimulation. To investigate the activation of the JAK2-STAT3 pathway induced by leptin, the duration of leptin stimulation was 2 h. In addition, for other changes induced by leptin, the duration of leptin stimulation was 24 h. The cells were harvested and used for the JAK2, P-JAK2, STAT3, P-STAT3, CD14, TLR4, MMP-13, and SOCS3 protein expression analyses by western blot. All experiments were performed 3 times.

### 2.8. Lentivirus Infection

Lentiviruses carrying SOCS3-shRNA and negative control were purchased from GenePharma (Shanghai, China). Primary chondrocytes were infected with lentiviruses-shSOCS3 (Lenti-shSOCS3) or lentiviruses-shSOCS3 negative control (NC) or untreated (CON) in culture media with polybrene. Cells displaying stable SOCS3 silencing were selected and cultured in DMEM/F12 medium containing puromycin. The expression of SOCS3 was verified by western blot. For the experiment, the CON, NC, and Lenti-shSOCS3 cells were stimulated with or without 200 ng/mL leptin for 2 h or 24 h before collected.

### 2.9. Statistical Analysis

Data was analyzed by SPSS software 20.0 (SPSS Inc., USA). All data were presented as mean ± SD. Independent-sample *t*-test was used to analyze the differences between the two groups. One-way analysis of variance (ANOVA), followed by Fisher's least significant difference (LSD) test, was used to analyze the differences among multiple groups. The correlation analyses were made with the Pearson correlation test. A difference with *P* < 0.05 was indicated statically significant.

## 3. Results

### 3.1. High-Fat Diet Induced Obesity and Increased Leptin Levels in Serum and Synovial Fluid in Rats

**Figure 1:**
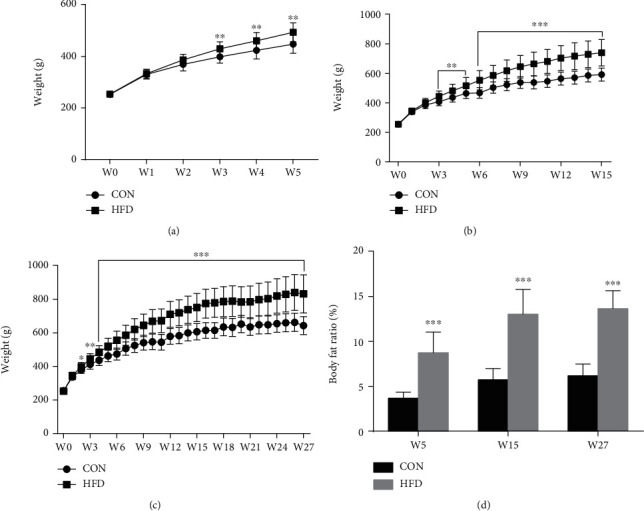
Effect of HFD on body weight and body fat ratio of rats. The curve of body weight was drawn in W5 (a), W15 (b), and W27 (c) by measuring body weight once a week. The body fat ratio was measured at the end of W5, W15, and W27 (d). Data were presented as mean ± SD, *n* = 12. An independent sample *t*-test was used to conduct statistical significance. ^∗∗^*P* < 0.01 and ^∗∗∗^*P* < 0.001 versus the CON group.

**Figure 2:**
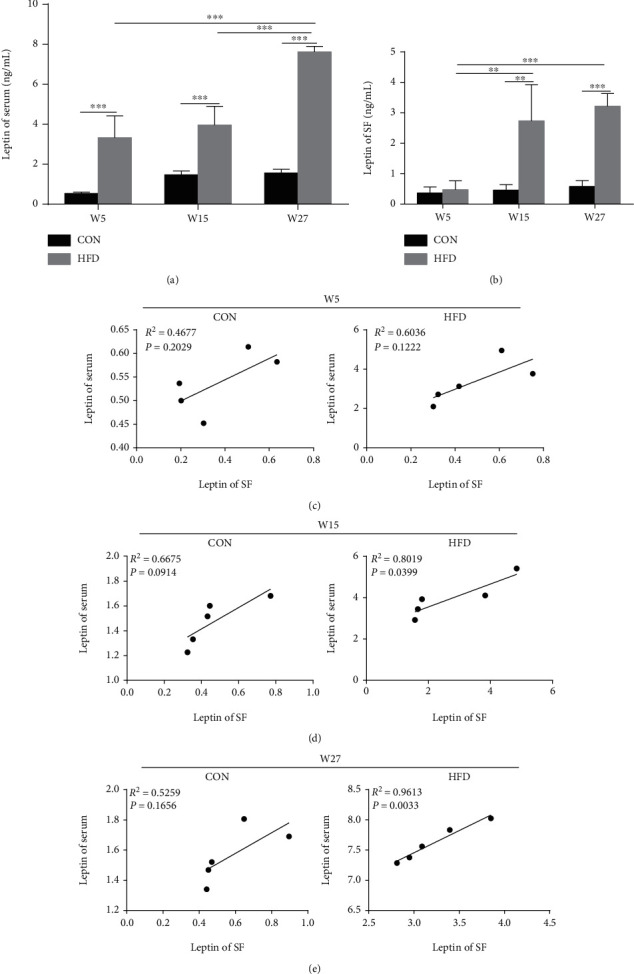
HFD increased leptin levels in the serum and SF in rats. The concentration of leptin in the serum (a) and SF (b) were detected by ELISA. Data were presented as mean ± SD, *n* = 5. Independent sample *t*-test and one-way ANOVA were used to conduct statistical significance. Pearson's correlation coefficient was used to calculate the correlation of leptin levels in the serum and SF at W5 (c), W15 (d), and W27 (e). Significant expression differences are shown as ^∗∗^*P* < 0.01 and ^∗∗∗^*P* < 0.001.

Begin with W3, the body weight of the HFD group was significantly higher than that of the CON group, and the difference of body weight between the HFD and CON groups increased over time (Figures 1(a)–1(c)). The body fat ratio was also increased in rats fed with HFD compared to the CON group (Figure 1(d)). As shown in Figure 2(a), HFD can increase the serum leptin levels in W5, W15, and W27 compared to the CON group. Moreover, with the increase of feeding time of HFD, the levels of leptin in the serum gradually increased. In SF, we observed that the increase of leptin levels in the HFD group was from W15 to W27, not from W5 compared to the CON group (Figure 2(b)). We also calculated the correlation of leptin levels in the serum and SF. In the CON group, there was no statistical correlation of leptin levels in the serum and SF at any time. For the HFD group, a significant positive correlation of leptin levels between the serum and SF was observed at W15 (*R*^2^ = 0.8019, *P* = 0.0399) and W27 (*R*^2^ = 0.9613, *P* = 0.0033) which is consistent with the result of Figure 2(b) (Figures 2(c)–2(e)).

### 3.2. Long-Term High-Fat Diet Could Induce Obesity-Related OA in Rats

**Figure 3:**
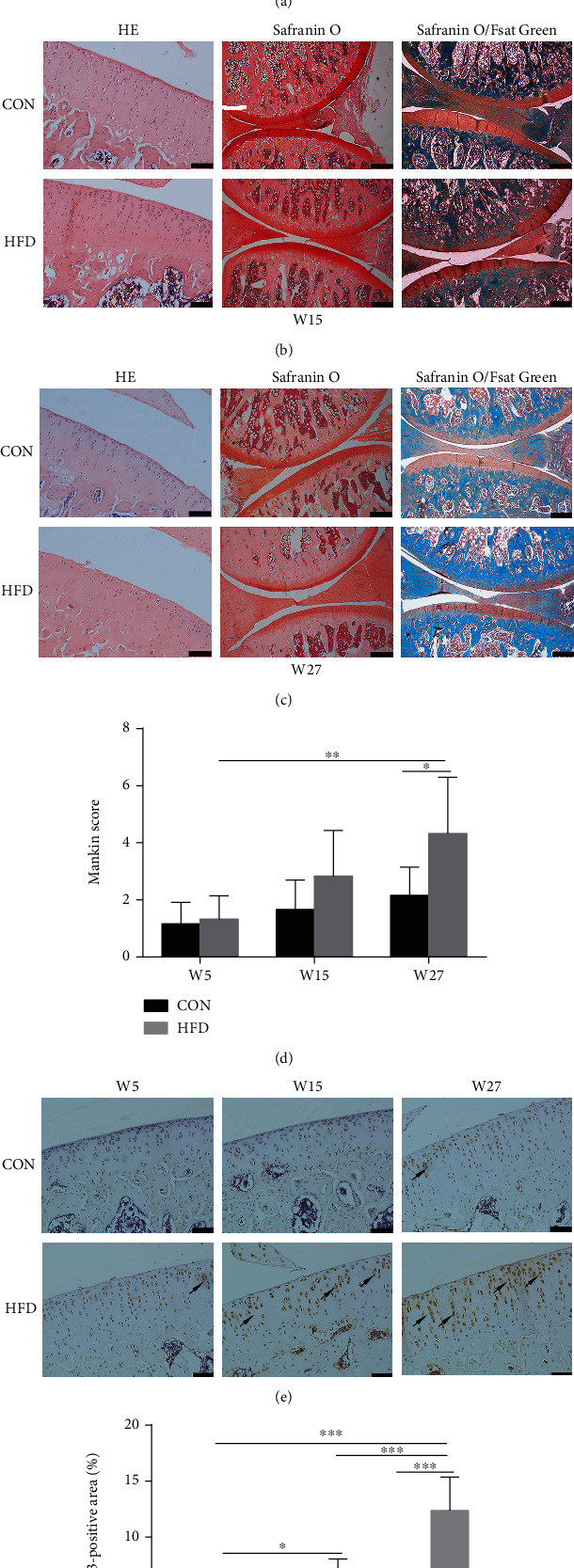
The effect of HFD on knee joints of rats. H&E staining (original magnification, 100x; scale bars = 100 *μ*m), Safranin O staining (original magnification, 40x; scale bars = 200 *μ*m), and Safranin O/Fast Green staining (original magnification, 40x; scale bars = 200 *μ*m) at W5 (a), W15 (b), and W27 (c). Mankin scores corresponding to the pathological changes of knee joint (d). Analysis of MMP-13-positive area (original magnification, 200x, scale bars = 50 *μ*m) was measured by immunohistochemical analyses (e, f) and represented by arrows. Data were presented as mean ± SD, *n* = 5. Independent sample *t*-test and one-way ANOVA were used to conduct statistical significance. Significant expression differences are shown as ^∗^*P* < 0.05, ^∗∗^*P* < 0.01, and ^∗∗∗^*P* < 0.001.

At W5 and W15, there were no obvious OA characteristics in the HFD group compared to the CON group (Figures 3(a) and 3(b)). At W27, the OA-like lesions were observed in the knee joints of HFD rats (Figure 3(c)). In addition, the Mankin scores of HFD rats increased gradually over time and higher than CON rats at W27 which is consistent with pathological staining results (Figure 3(d)). We also detected MMP-13 levels in knee joints by immunohistochemical analyses. The reason is that most MMPs are involved in the turnover of extracellular matrix and the associated destruction of the articular cartilage in OA [16]. Still, the soluble collagenase MMP-13 is crucial for this destruction to occur [17]. At W27, the MMP-13 levels in HFD rats were remarkably increased compared to CON rats. Moreover, the expression of MMP-13 in the HFD group increased significantly over time (Figures 3(e) and 3(f)).

### 3.3. High-Fat Diet Activated JAK2-STAT3 Signaling Pathway in Knee Articular Cartilage of Rats

**Figure 4:**
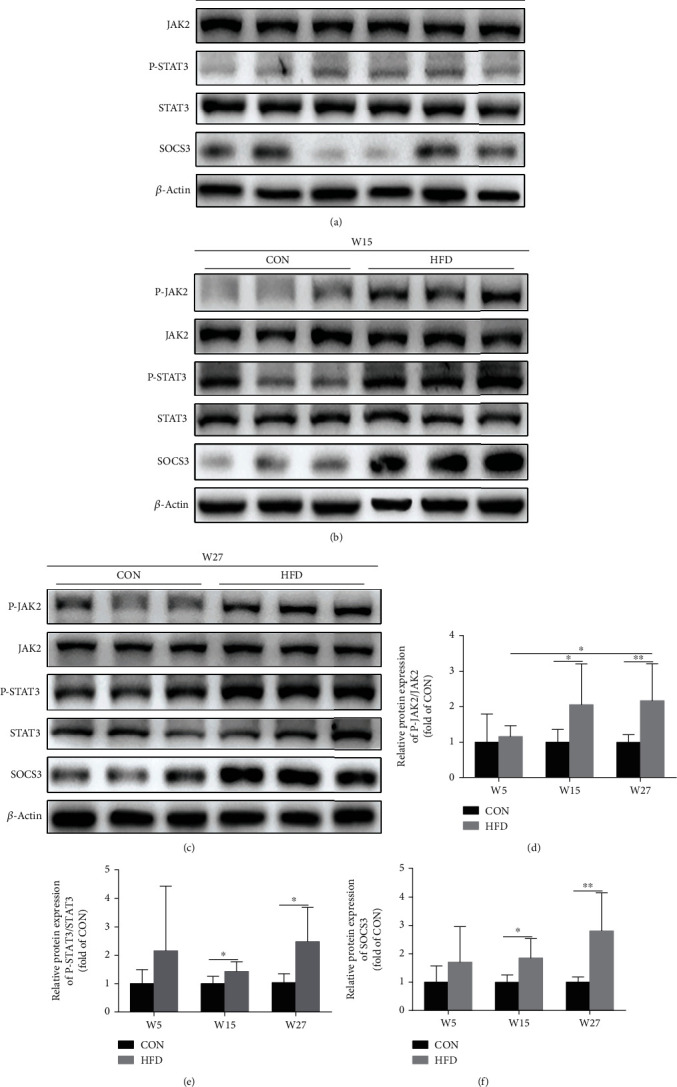
HFD activated the JAK2-STAT3 signaling pathway in the knee articular cartilage of rats. Western blot analysis was used to detect JAK2-STAT3 signaling and SOCS3 protein expression at W5 (a), W15 (b), and W27 (c), and the corresponding bar graph data of JAK2-STAT3 signaling (d, e) and SOCS3 (f) were presented as mean ± SD, *n* = 7. Independent sample *t*-test and one-way ANOVA were used to conduct statistical significance. Significant expression differences are shown as ^∗^*P* < 0.05 and ^∗∗^*P* < 0.01.

As shown in Figure 4, HFD increased the expression of P-JAK2 and P-STAT3 at W15 and W27 compared to the CON group which indicated that HFD activated the JAK2-STAT3 pathway (Figures 4(b)–4(e)). Additionally, SOCS3, the negative regulator of leptin signaling, was also elevated in the HFD group compared to the CON group at W15 and W27 (Figures 4(b), 4(c), and 4(f)).

### 3.4. High-Fat Diet Upregulated CD14/TLR4 Expression in Knee Articular Cartilage of Rats

**Figure 5:**
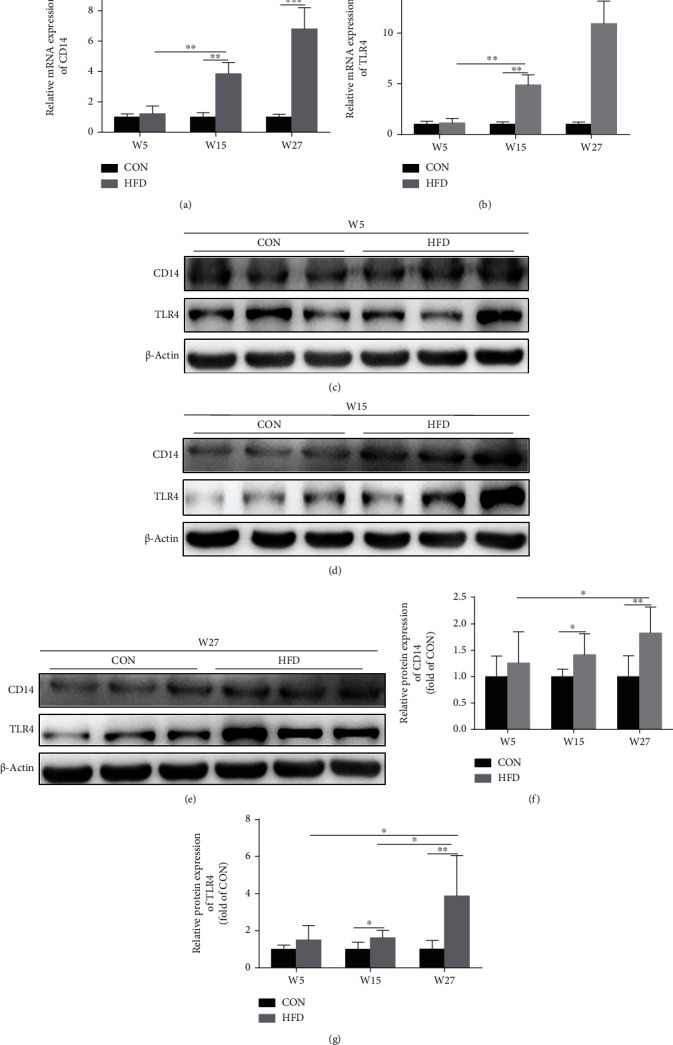
HFD upregulated CD14/TLR4 expression in the knee articular cartilage of rats. The mRNA expression of CD14 and TLR4 was measured by RT-PCR (a, b). Western blot analysis was used to detect CD14 and TLR4 protein expressions at W5 (c), W15 (d), and W27 (e), and the corresponding bar graph data of JAK2-STAT3 signaling (f, g) were presented as mean ± SD, *n* = 7. Independent sample *t*-test and one-way ANOVA were used to conduct statistical significance. Significant expression differences are shown as ^∗^*P* < 0.05 and ^∗∗^*P* < 0.01.

HFD increased CD14 and TLR4 mRNA expressions compared to the CON group at W15 and W27 (Figures 5(a) and 5(b)). In line with the result of mRNA expression, the western blot analysis also showed that the protein expressions of CD14 and TLR4 in the HFD group were elevated at W15 and W27 compared to the CON group (Figures 5(c)–5(g)).

### 3.5. Leptin Regulated CD14 and TLR4 as well as SOCS3 and MMP-13 Expressions through JAK2-STAT3 Pathway in Chondrocytes

**Figure 6:**
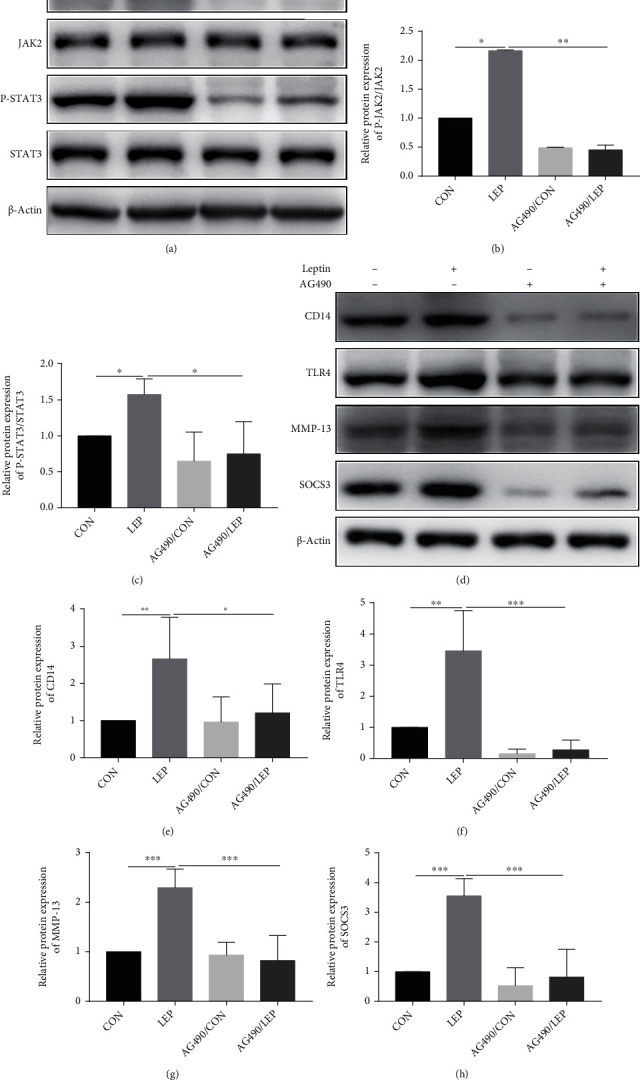
Leptin regulated CD14 and TLR4 as well as SOCS3 and MMP-13 expressions through the JAK2-STAT3 pathway in chondrocytes. The protein expression of JAK2-STAT3 signaling, SOCS3, CD14, TLR4, and MMP-13 were measured by western blot analysis (a, d). The bar graph summarized data of blots for JAK2-STAT3 signaling (b, c), CD14 (e), TLR4 (f), MMP-13 (g), and SOCS3 (h). Data were presented as mean ± SD, *n* = 3. Independent sample *t*-test and one-way ANOVA were used to conduct statistical significance. Significant expression differences are shown as ^∗^*P* < 0.05, ^∗∗^*P* < 0.01, and ^∗∗∗^*P* < 0.001.

To verify the relationship between CD14/TLR4 and the JAK2-STAT3 pathway, the specific inhibitor AG490 of JAK2-STAT3 was used to treat chondrocytes. We observed that activation of the JAK2-STAT3 signaling pathway by leptin can be blocked by AG490 (Figures 6(a)–6(c)). The protein expression of CD14 and TLR4 was downregulated after leptin treatment with AG490 compared to that without AG490. We also examined SOCS3 and MMP-13 protein expressions and found that they were also downregulated after leptin treatment in the presence of AG490 (Figures 6(d)–6(h)).

### 3.6. Silencing SOCS3 Enhanced Leptin-Induced JAK2-STAT3-CD14/TLR4 Activation in Chondrocytes

**Figure 7:**
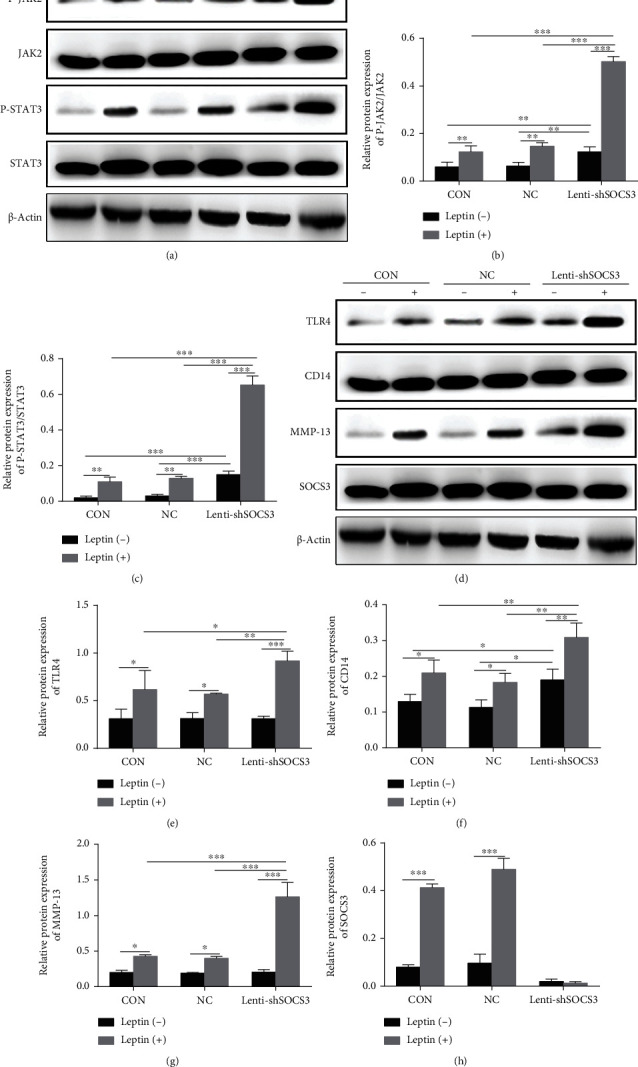
Silencing SOCS3 enhanced leptin-induced JAK2-STAT3-CD14/TLR4 activation in chondrocytes. The protein expressions of JAK2-STAT3 signaling, SOCS3, CD14, TLR4, and MMP-13 were measured by western blot analysis (a, d). The bar graph summarized data of blots for JAK2-STAT3 signaling (b, c), CD14 (e), TLR4 (f), MMP-13 (g), and SOCS3 (h). Data were presented as mean ± SD, *n* = 3. Independent sample *t*-test and one-way ANOVA were used to conduct statistical significance. Significant expression differences are shown as ^∗^*P* < 0.05, ^∗∗^*P* < 0.01, and ^∗∗∗^*P* < 0.001.

SOCS3 is known as a negative feedback regulatory protein in the leptin signaling pathway. Here, we found that the activation of the JAK2-STAT3 pathway was enhanced after SOCS3 knockdown (Figures 7(a)–7(c)). Moreover, the protein expressions of CD14 and TLR4 which are the downstream protein of the JAK2-STAT3 pathway were also significantly increased after silencing SOCS3 (Figures 7(d)–7(h)).

## 4. Discussion

Obesity-related OA has been considered to be caused by the mechanical effects of obese individuals on joint load and systemic inflammatory factors [18], while the pathogenesis of obesity-related OA has yet to be defined. In our study, we found that rats on a HFD had higher body weight and body fat percentage. Rats fed a HFD have been observed to have increased Mankin scores and upregulated MMP-13 expression in the articular cartilage which is in accordance with histological staining results. It further indicated that HFD could lead to OA-like changes which are in accordance with our previous reports [7, 15].

Adipokine leptin is a substance mainly secreted by adipocytes. It can inhibit feeding and increase thermogenesis through hypothalamic receptors [19]. In addition, it may also act as an important role in the pathogenesis of OA [20–23]. In the present study, HFD rats, in comparison to CON rats, showed higher leptin level in serum, but not in SF at the beginning (W5). However, a higher level of leptin in both the serum and SF was shown at 15 weeks and 27 weeks, which revealed that the serum leptin by a HFD changed at a very early age (W5), while the changes of SF leptin was later. It is reported that plasma and SF leptin were significantly correlated in juvenile idiopathic arthritis [24]. In the present study, there was also a positive correlation of leptin levels between the serum and SF at W15 and W27 when the OA characteristic has emerged. In general, leptin in SF is mainly derived from synovial cells, infrapatellar fat pad, and articular chondrocytes [25]. The increased level of leptin in circulation can lead to systemic low-grade inflammation [26, 27] and account for the increased permeability of an inflamed synovial membrane [28, 29]. Accordingly, the serum leptin could get into SF, demonstrating the increase of serum leptin level is prior to that of SF in the development of OA. Leptin levels were increased before the OA changes, indicating that leptin may be a triggering factor for obesity-related OA. Besides, we tested the IL-1*β* level in both the serum and SF as well, which is because the elevation of IL-1*β* is a key manifestation for inflammation. However, in the present study, it failed to measure because most of the levels were beyond the detecting range of the kit, which raises an important point that leptin may be a more sensitive indicator than IL-1*β* in HFD-induced obesity-related OA rats. Interestingly, at the 27 W time point, the number of samples with detectable IL-1*β* levels (data not shown) in the HFD group was higher than that in the CON group in both the serum and SF, suggesting the OA-promoting effect of HFD.

TLR4, a modulator of innate immunity, contributes to obesity-related OA pathogenesis via mediating metabolic inflammation and cartilage catabolism in OA joints [30]. In the extremely obese leptin- or leptin receptor-deficient mice, leptin induces TLR responses and promotes OA [4, 31], implying that leptin-linked OA was related to the activation of TLR4. Studies have demonstrated a role of leptin on regulating the JAK2-STAT3 signaling pathway, and STAT3 was known to have an essential role on inflammatory cytokine production [32, 33]. In the present study, we found that the JAK2-STAT3 pathway was significantly activated by HFD in the cartilage in the later stage, which might be due to the increased expression of leptin. Previous studies reported that leptin increased CD14 expression via STAT3 signaling, and CD14 is a coreceptor for TLR4-induced proinflammatory response [34, 35]. Our study found that the CD14 and TLR4 expressions were elevated with HFD treatment. Also, leptin exhibited the same effect on chondrocytes as *in vivo* study that the JAK2-STAT3 and its downstream targets expression were upregulated. AG490, as a JAK2 inhibitor, was reported to significantly ameliorate leptin-induced apoptosis in damaged chondrocytes [36]. Our study showed that with the presence of AG490, leptin could not affect the expression of TLR4 and other inflammatory-related proteins which suggested that leptin may regulate TLR4 expression through the JAK2-STAT3 pathway in obesity-related OA.

SOCS3 mediates feedback inhibition of the leptin signaling in the hypothalamus [37]; however, our previous findings demonstrated that negative feedback regulation of SOCS3 in leptin signaling is weaker in the articular cartilage [15]. It indicated that the tissue-specific regulation of SOCS3 in leptin signaling may exist between CNS and peripheral tissues [38]. Leptin did have an effect on TLR4 through the JAK2-STAT3-CD14 pathway, and the increased SOCS3 expression did not perform a significant inhibiting effect on leptin signaling in our present study. Then, in *in vitro* experiments, we knocked down SOCS3 in order to activate the JAK pathway in disguise. Interestingly, after silencing SOCS3, TLR4 expression was not affected without leptin treatment, indicating that SOCS3 had no direct interaction with TLR4. However, after leptin was administered, the expression of TLR4 was increased. Combined with the previous (Figure 6) results, it proved that leptin can indeed regulate the expression of TLR4, and at least partly through the JAK signaling pathway. The upregulation of MMP-13 also demonstrated that degradation of chondrocytes was increased by leptin, suggesting that the chondrocytes were more sensitive to leptin after SOCS3 silencing.

## 5. Conclusions

**Figure 8:**
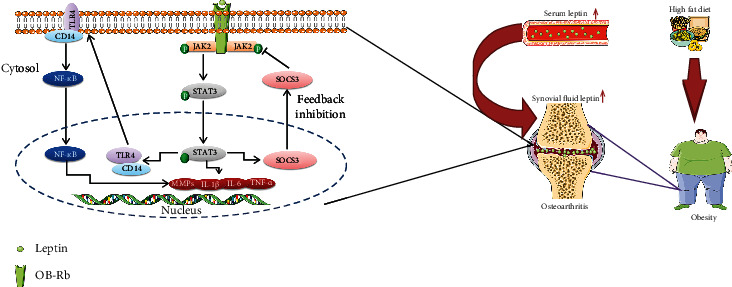
SOCS3 negatively regulates leptin signaling and its mechanism of the TLR4 signaling pathway.

In summary, our study concluded that the increase of serum leptin level may be an initiating factor of obesity-related OA, which further elevated the level of leptin in SF and induce the JAK2-STAT3 signaling pathway to promote the development of OA. TLR4 is at least partially regulated by leptin through the JAK2-STAT3-CD14 pathway. SOCS3 acts as a negative feedback inhibitor of leptin signaling, and enhancing its expression may help to inhibit the progression of OA (Figure 8). Therefore, the potential therapeutic strategy for obesity-related OA could be by inhibiting the leptin signal via the reduction of leptin levels and enhancement of SOCS3 expression, or by directly suppressing TLR4 expression.

## Data Availability

The data used to support the findings of this study are available from the corresponding author upon request.
